# A Germline Polymorphism of DNA Polymerase Beta Induces Genomic Instability and Cellular Transformation

**DOI:** 10.1371/journal.pgen.1003052

**Published:** 2012-11-08

**Authors:** Jennifer Yamtich, Antonia A. Nemec, Agnes Keh, Joann B. Sweasy

**Affiliations:** Departments of Therapeutic Radiology and Genetics, Yale University School of Medicine, New Haven, Connecticut, United States of America; Baylor College of Medicine, United States of America

## Abstract

Several germline single nucleotide polymorphisms (SNPs) have been identified in the *POLB* gene, but little is known about their cellular and biochemical impact. DNA Polymerase β (Pol β), encoded by the *POLB* gene, is the main gap-filling polymerase involved in base excision repair (BER), a pathway that protects the genome from the consequences of oxidative DNA damage. In this study we tested the hypothesis that expression of the *POLB* germline coding SNP (rs3136797) in mammalian cells could induce a cancerous phenotype. Expression of this SNP in both human and mouse cells induced double-strand breaks, chromosomal aberrations, and cellular transformation. Following treatment with an alkylating agent, cells expressing this coding SNP accumulated BER intermediate substrates, including single-strand and double-strand breaks. The rs3136797 SNP encodes the P242R variant Pol β protein and biochemical analysis showed that P242R protein had a slower catalytic rate than WT, although P242R binds DNA similarly to WT. Our results suggest that people who carry the rs3136797 germline SNP may be at an increased risk for cancer susceptibility.

## Author Summary

Cancer is the second leading cause of death in the United States. The maintenance of genomic integrity is dependent on faithful DNA replication and repair. The base excision repair (BER) pathway is responsible for repairing at least 20,000 lesions per cell per day. DNA polymerase beta (Pol β) is the main polymerase in the BER pathway and is mutated in up to 40% of human tumors. However, little is known regarding any germline mutations found in the human population. Here, we provide evidence that the germline variant of Pol β, P242R, has a slower catalytic rate than the wild-type Pol β. This reduced rate induces an increase in chromosomal aberrations, a type of genomic instability that can lead to cancer. We also show that expressing P242R in human cells induces cellular transformation, anchorage-independent growth, and an increased rate of proliferation: all hallmarks of tumorigenesis. Together, our data suggest that the germline Pol β variant can drive carcinogenesis.

## Introduction

DNA Polymerase β (Pol β) is the main polymerase involved in the base excision repair pathway (BER), the pathway responsible for repairing up to 20,000 endogenous lesions per cell per day [1], [2]. Pol β is a bifunctional polymerase, containing both deoxyribose phosphate (dRP) lyase and nucleotidyl transferase activities (reviewed in [3]). One or both of these activities are essential, as Pol β knockout mice die shortly after birth [4].

Two germline SNPs of the *POLB* gene (rs12678588and rs3136797) have been previously identified, and the variant alleles have been shown to be present in specific populations [5], [6]. The rs12678588 SNP results in a nonsynonymous amino acid substitution of glutamine for arginine at residue 137 (R137Q). In the wild-type (WT) protein, Arg137 is methylated by the protein arginine N-methyltransferase 1 (PRMT1), leading to a reduction in proliferating cell nuclear antigen (PCNA) binding [7]. R137Q is a slow polymerase with decreased BER activity in cell extracts, and cells expressing this variant have increased formation of AP sites following methyl methanesulfonate (MMS) exposure [8]. Little is known about the biochemical and cellular characteristics of the rs3136707 SNP, in which the proline at residue 242 is altered to arginine (P242R) or its role in human health. Carriers of this allele include populations from Eastern Europe [6]. Interestingly, patients heterozygous for this allele exhibited decreased survival when treated for either lung cancer or lymphoma [9], [10]. Additionally, this residue is located at the base of Loop II, a region that has been shown by us to be critical for polymerase activity and fidelity [11]–[13].

In this study, we tested the hypothesis that the P242R germline *POLB* variant has a functional phenotype that could drive carcinogenesis. We found that expression of a cDNA encoding the P242R protein in both human and mouse cells induce chromosomal aberrations and cellular transformation. We also show that purified P242R protein is a slow polymerase that binds DNA tightly. In combination, our results suggest that cells expressing P242R accumulate BER intermediates that result in the induction of DSBs and chromosomal aberrations that lead to cellular transformation. Our results also indicate that the P242R germline variant of Pol β could result in aberrant BER in carriers of the allele, potentially leading to increased cancer predisposition.

## Results

### Expression of P242R Induces Chromosomal Aberrations

**Figure 1:**
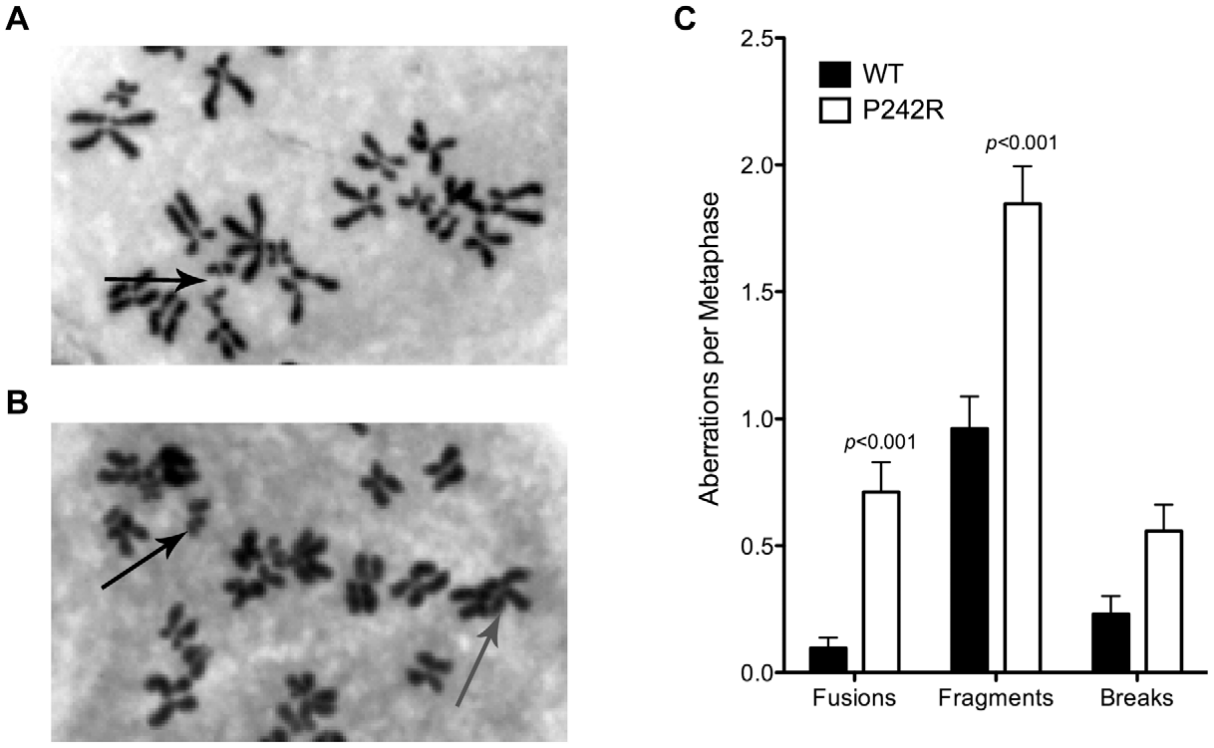
Chromosomal aberrations in P242R-expressing cells. Representative metaphase spread of MCF10A expressing (A.) WT or (B.) P242R Pol β. Chromosomal fusions are shown with the gray arrow and fragments are shown with black arrows. C. Number of aberrations per metaphase. A total of at least 50 metaphases were scored for each cell line.

Previous work on Pol β has shown that expression of certain tumor-specific single amino acid variants can induce genomic instability in the form of chromosomal aberrations [14], [15]. Therefore, we characterized chromosomal aberrations in MCF10A normal human epithelial cells expressing the germline variant P242R. We generated stable MCF10A cell lines expressing C-terminally HA-tagged Pol β-WT or P242R at equal levels to the endogenous WT protein (Figure S1A) and scored metaphase spreads from each of these lines. Figure 1A–1B shows examples of metaphase spreads from MCF10A cells expressing WT or P242R Pol β, respectively. MCF10A cells expressing P242R had increased amounts of chromosomal fusions and fragments compared to cells expressing the WT (Figure 1C). To determine if P242R was a mutator in cells, we performed the λ*cII* forward mutation assay using a C127λb clonal cell line as we describe [16]. Briefly, these cells carry the λ genome and express either P242R or WT Pol β. The phage λ genome is packaged from isolated genomic DNA and the mutant frequency is obtained by infection of *E. coli* using differential plating conditions as we describe [16]. The spectra of mutations induced by P242R and WT Pol β are generated by sequencing purified plaques. We found that expression of P242R does not induce an increased frequency of point mutations nor a mutation spectrum different from that of WT Pol β, suggesting that it is not a mutator polymerase (Table S1 and Figure S2). Therefore, our results suggest that expression of P242R induces genomic instability in the form of chromosomal aberrations.

### Single-Strand and Double-Strand Break Accumulation in P242R-Expressing Cells

**Figure 2:**
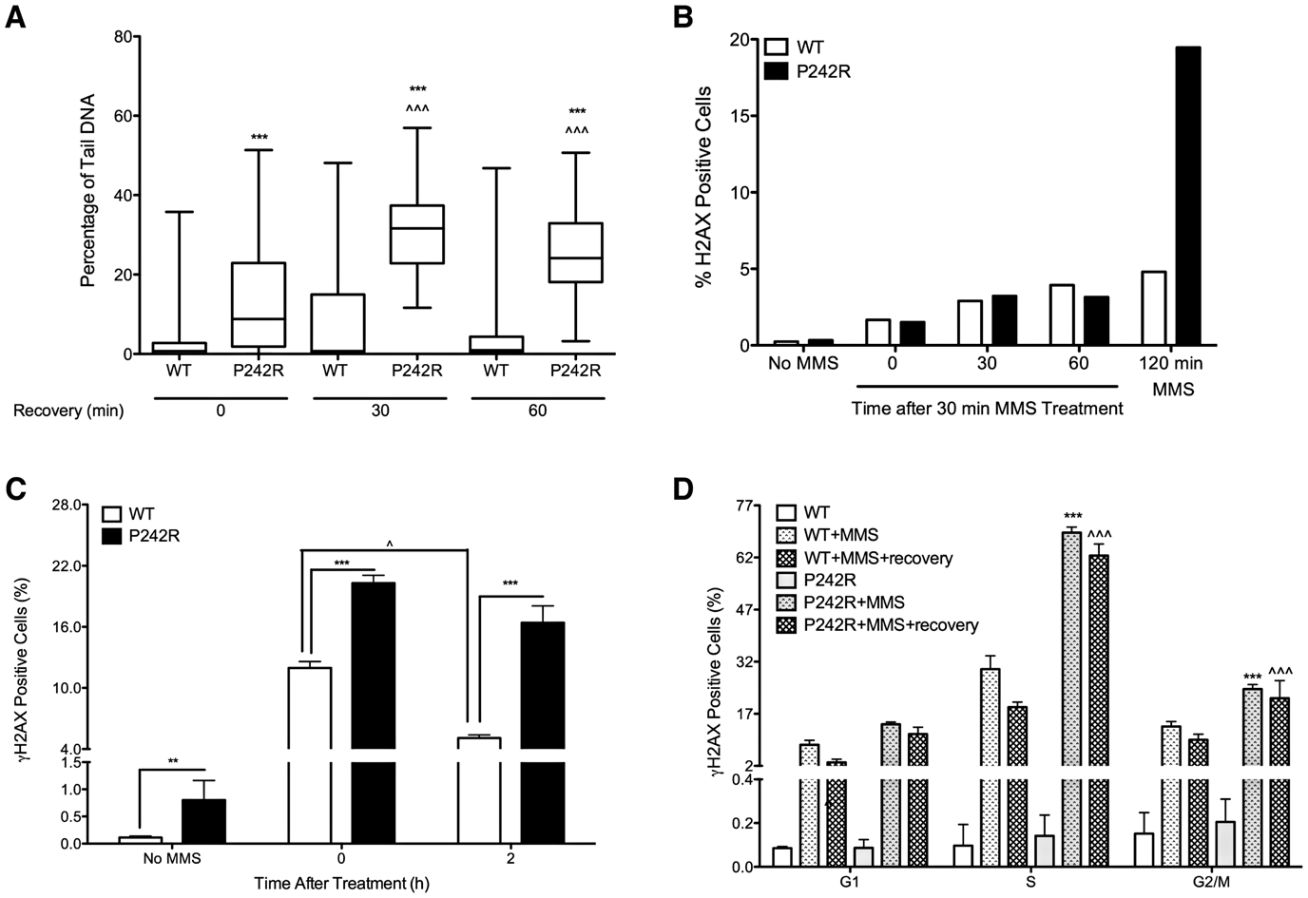
Accumulation of BER intermediates in MCF10A cells expressing P242R Pol β. A. MCF10A pools expressing WT or P242R Pol β were treated with 2 mM MMS for 30 minutes and allowed to recover for 0, 30, or 60 min and single-strand breaks (SSBs) were analyzed by comet assay. The percentage of tail DNA is plotted on the Y-axis. B. MCF10A pools expressing WT or P242R Pol β were treated with 2 mM MMS for 30 min and allowed to recover for 0, 30, or 60 min, stained with γH2AX antibody, and analyzed by flow cytometry. Cells were treated for 120 min as a positive control. C–D. MCF10A pools expressing WT or P242R Pol β were treated with 2 mM MMS for 2 h and allowed to recover for 0 or 2 hours. Cells were stained with γH2AX antibody and propidium iodide to assess the levels of double-strand breaks (DSBs) and the cell cycle phase, respectively, and analyzed by flow cytometry. Data are plotted as the mean ± SEM. Data are plotted as the mean ± SEM (n = 3). A and C. ** and *** denote *p*<0.01 and 0.001, respectively. ∧ denotes *p*<0.05 comparing 0 vs 2 h recovery within each cell line. ∧∧∧ denotes *p*<0.001 comparing 30 or 60 min recovery to 0 recovery. D. *** denotes *p*<0.001 comparing WT+MMS to P242R+MMS in each phase of the cell cycle. ∧ and ∧∧∧ denote *p*<0.05 and 0.001, respectively, comparing WT+MMS+recovery to P242R+MMS+recovery in each phase of the cell cycle.

A key role of Pol β is to fill gaps that arise from excision of DNA damage during BER. Chromosomal aberrations are known to arise from breaks in the DNA and aberrant BER can lead to the accumulation of DNA breaks [17]. Therefore, we wished to determine whether treatment with the alkylating agent methylmethane sulfonate (MMS) induces the formation of SSB and DSB BER intermediates in MCF10A cells expressing P242R. MMS induces DNA base damage that is repaired by the BER pathway and therefore treatment of cells with MMS leads to an increase in substrates recognized by Pol β. We used the alkaline comet assay to quantify SSBs induced by MMS. Following treatment with MMS for 30 min, cells expressing P242R have a significantly higher level of SSBs than cells expressing WT Pol β (Figure 2A) (*p*<0.001). Even after cells were allowed to recover from the MMS treatment for 30 or 60 min, greater levels of SSBs were still observed in P242R cells compared to WT cells (*p*<0.001). In fact, the percentage of tail DNA was increased in P242R cells allowed to recover compared to P242R cells treated with MMS for 30 min without any recovery (*p*<0.001) suggesting that cells expressing P242R were unable to efficiently repair the damage or were continuing to accumulate damage. Although the alkaline comet assay measures SSBs, DSBs could also be present. To determine if this was the case, we treated cells with MMS for 30 min and allowed them to recover as in the comet assay, except we monitored γH2AX staining as an indicator of DSB formation. Two to about five percent of cells expressing either WT or P242R exhibited DSBs when treated with MMS for 30 followed by a recovery period for 0, 30 or 60 min. (Figure 2B). Because the levels of DSBs are similar in cells expressing WT or P242R using this protocol, our results suggest that the comet assay is not predominantly detecting DSBs. Importantly, DSBs are not observed to accumulate in P242R-expressing cells unless they are treated for at least 2 hrs with MMS. We then treated the MCF10A pools expressing WT or P242R with MMS for two hours and analyzed γH2AX staining as an indicator of DSB formation. We also stained with propidium iodide for cell cycle analysis. We found that exposure to MMS for 2 hours increased the levels of DSBs in both WT and P242R-expressing cells although MMS induced significantly more DSBs in P242R cells compared to WT Pol β (Figure 2C) (*p*<0.001). Moreover, cells expressing WT Pol β began to repair the damage after 2 hours following treatment (*p*<0.05) whereas cells expressing P242R continued to exhibit γH2AX staining, suggesting that these cells continued to accumulate DSBs longer and/or had delayed repair compared to cells expressing WT Pol β. In fact, WT Pol β expressing cells are able to repair the damage after 2–4 hours and return to background levels of γH2AX staining whereas even after a 4 hour recovery, γH2AX positive foci were observed in cells expressing P242R Pol β (Figure S3). Strikingly, we also observe a significant increase in γH2AX staining in untreated cells expressing P242R Pol β compared to WT (*p*<0.05) (Figure 2C). The differences observed were not due to varying levels of expression as western analysis shows that WT and P242R were expressed at similar levels in these cells (Figure S1A). Furthermore, although γH2AX positive cells were observed in all three phases of the cell cycle, cells expressing P242R had a significantly higher percentage of γH2AX cells in both S and G2/M phases (Figure 2D) (*p*<0.001) suggesting that the DSBs may be formed, in part, during DNA replication.

### Expression of P242R Pol β in Mouse and Human Cells Induces Cellular Transformation

**Figure 3:**
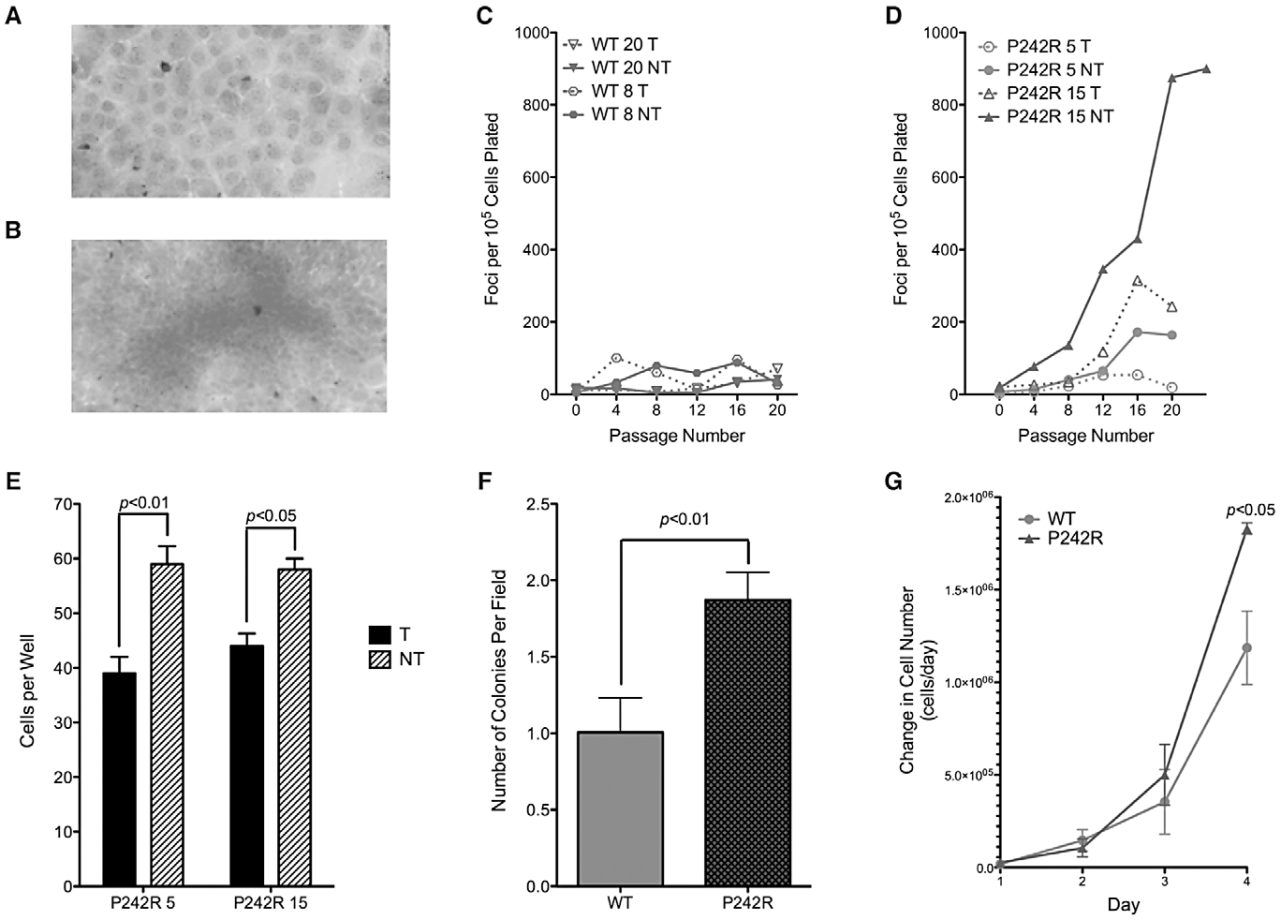
Expression of P242R induces cellular transformation. A. Representative image of crystal violet stained untransformed cells. Note the clear monolayer of cell growth. B. Representative image of a focus. Note the accumulation of cells as marked by dark staining. C–D. Focus formation assay with two clonal cell lines either inducing (NT, solid line) or not inducing (T, dashed line) exogenous (C.) WT or (D.) P242R Pol β. Mean foci per 10^5^ cells plated (± standard deviation of plating replicates) plotted against passage number. E. Anchorage independent growth assay with P242R clonal cell lines from the focus formation assay. Cells per field (± standard error) are plotted on the Y-axis. F. Anchorage independent growth assay with MCF10A pools expressing WT or P242R. The number of colonies per field are plotted on the Y-axis. G. WT or P242R MCF10A pools were plated in 5 dishes at a density of 25,000 or 50,000 cells per dish. Cells were trypsinized and counted each day. Data are plotted as the mean ± SEM of the change in cell number (n = 3).

We tested the hypothesis that the genomic instability resulting from expression of the germline Pol β P242R protein induces cellular transformation. We generated clonal C127λb cell lines expressing exogenous HA-tagged human Pol β (WT and P242R) at approximately equal levels to endogenous Pol β in a tetracycline-repressible manner (Figure S1B). In the focus formation assay, untransformed cells will grow to confluence forming a monolayer (Figure 3A), while transformed cells will continue to grow after reaching confluence, forming foci (Figure 3B). Expression of P242R Pol β induced cellular transformation whereas expression of WT Pol β did not (Figure 3C–3D). To confirm transformation in these lines, we used a soft agar growth assay in which transformed cells that are capable of anchorage-independent growth will grow when plated on soft agar, while non-transformed cells will not. This assay confirms the results of the focus formation assay, showing that expression of P242R induces anchorage independent growth (Figure 3E) (*p*<0.01 and 0.05 for P242R clones 5 and 15, respectively). Next, we assessed anchorage independent growth in the human MCF10A cells. Similar to the C127λb clonal lines, MCF10A pools expressing P242R also displayed increased numbers of cells able to grow in soft agar compared to WT (Figure 3F) (*p*<0.05). Additionally, MCF10A cells expressing P242R had an increased rate of proliferation, another hallmark of cancer cells (Figure 3G) (*p*<0.05). Together, these data suggest that expression of Pol β P242R induces cellular transformation in both mouse and human cells.

### Expression of P242R in WT MEFs Does Not Confer Sensitivity to MMS

**Figure 4:**
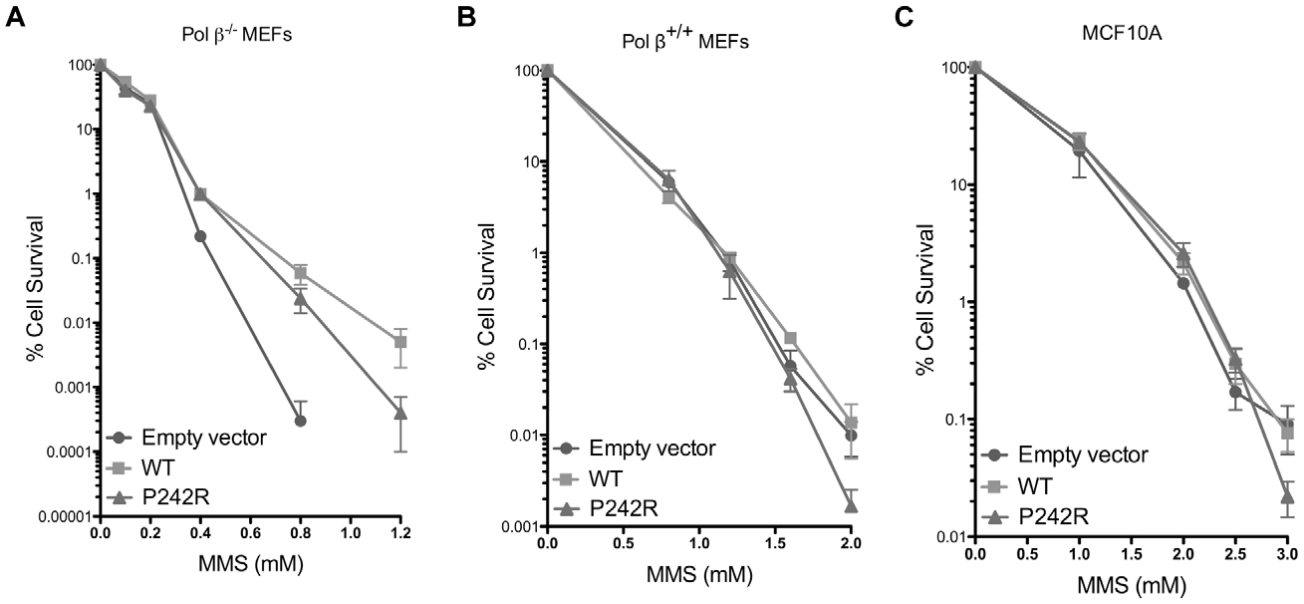
P242R Pol β confers slight sensitivity to MMS compared to WT. Clonogenic survival assays were conducted with (A.) Pol β^−/−^ MEFs, (B.) Pol β^+/+^ MEFs, or (C.) MCF10A pools expressing WT or P242R Pol β. Filled circles represent results from pools expressing empty vector, filled squares represent pools expressing WT Pol β, and filled triangles represent pools expressing P242R Pol β. Data are plotted as the mean ± SEM (n = 3).

The accumulation of BER intermediates suggests that P242R may not function in the gap-filling step as well as WT Pol β. Expression of P242R in the Pol β^−/−^ mouse embryonic fibroblasts (MEFs) partially rescued cellular survival in response to treatment with MMS, albeit not as well as expressing WT Pol β (Figure 4A). The reduced ability of P242R to rescue cells from the effects of MMS compared to WT implies that P242R has a partially impaired BER function. However, the rs3136797 SNP, encoding P242R, is present as a heterozygous allele and rarely as a homozygous allele [6]. In addition, the cell lines we employed for our studies, namely, C127λb and MCF10A, both express WT Pol β. Therefore, we investigated whether expression of the P242R variant in the presence of WT Pol β sensitizes cells to MMS, as has been shown for the polymerase-dead E295K variant [15]. We conducted clonogenic survival assays using pools of Pol β^+/+^ MEFs and MCF10A cells expressing either WT or P242R Pol β or empty vector. Expression of P242R only slightly sensitized cells to high concentrations of MMS in a Pol β proficient background (Figure 4B–4C). In combination with our chromosomal aberration studies, this suggests that some of the cells harboring genomic instability are likely to survive and could become transformed.

### The P242R Germline Variant Is a Slow Polymerase

**Figure 5:**
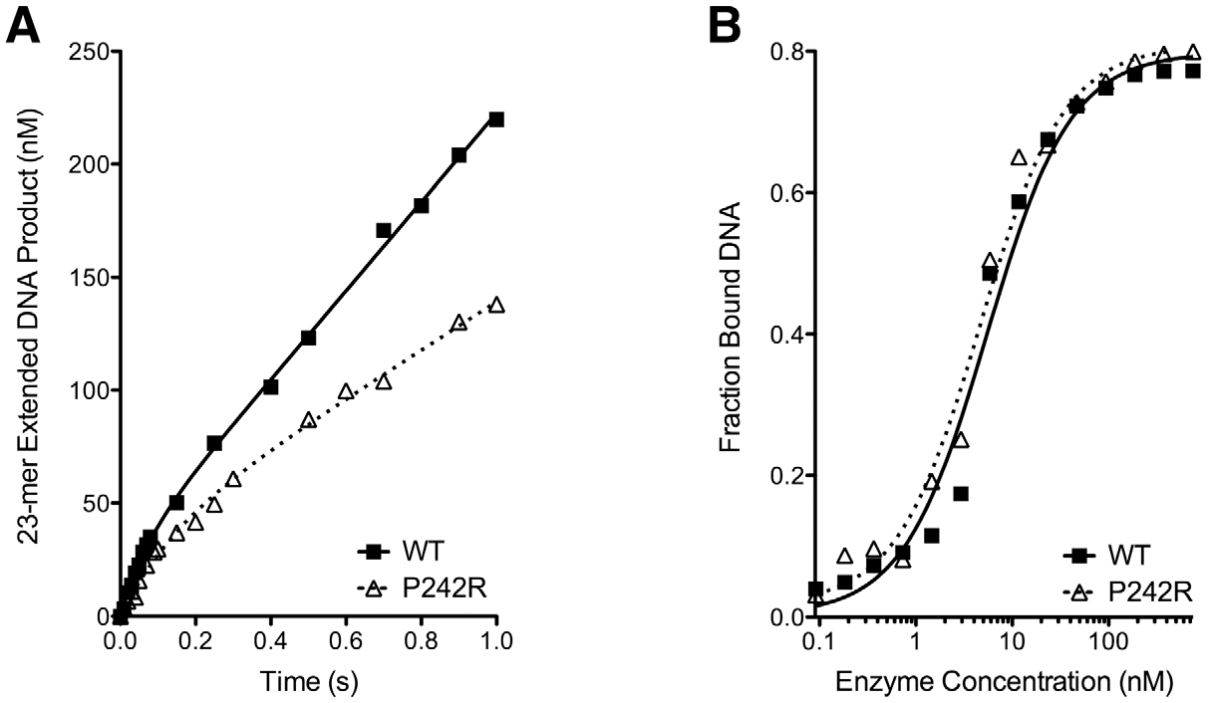
The P242R germline variant of Pol β is slow and binds DNA tightly. A. Representative results from a presteady-state burst assay. Results for the WT are shown as filled squares fit with a solid curve. Results for the P242R are shown as open triangles fit with a dashed curve. The assay was repeated four times for each protein. B. Representative results from a gel electrophoretic mobility shift assay. Results for the different proteins are shown as in A.

The recombinant WT and P242R proteins were purified from *E. coli* and studied in a presteady-state burst assay. This assay used a radiolabeled 1 bp gapped DNA substrate, the preferred substrate for Pol β. Both proteins fit a biphasic burst of product formation, typical of Pol β activity (Figure 5A). However, P242R had decreased initial burst (*k*_obs_ = 14±2 and 7.5±0.2 sec^−1^, WT and P242R, respectively) and steady-state rate compared to WT (*k*_ss_ = 3.3±0.5 and 1.7±0.2 sec^−1^, WT and P242R, respectively). This decreased activity was not due to decreased DNA binding, as the gel electrophoretic mobility shift assay showed that the P242R variant binds 1-bp gapped DNA with similar affinity to WT (K_d_ = 5±1 and 5±1 nM, WT and P242R, respectively) (Figure 5B). Together, our data suggest that the slow rate of DNA synthesis catalyzed by P242R could result in unfilled gaps that lead to genomic instability and cellular transformation.

## Discussion

Previous work from our laboratory showed that the rs3136797 SNP is present at maximum frequencies of 2.4% and is predominantly found in Eastern European populations and Keralites [6]. This polymorphism is one of two in the *POLB* gene that results in a missense mutation and the allele with the rs3136797 SNP encodes the P242R Pol β protein. Using haplotype analysis, this allele was shown to be evolutionarily distinct from other *POLB* SNPs studied and its low frequency suggests that few homozygotes exist. Given that Pol β plays a critical role in the repair of endogenous DNA damage and functions to maintain genome stability, we tested the hypothesis that the P242R germline variant has a functional phenotype related to carcinogenesis. Our work shows that expression of P242R Pol β in the presence of WT Pol β induces genomic instability and cellular transformation in both mouse and human cells. Our results are consistent with the interpretation that the people who carry the P242R Pol β SNP are at increased risk for cancer.

### P242R Is Located in a Region of Pol β That Is Critical for Activity

Pro242 of Pol β is located at the base of Loop II, a structure that we previously showed to be important for Pol β activity and fidelity [11], [12]. Loop II is highly flexible, solvent exposed, and quite far away from the active site of Pol β. Mutations in amino acids within this loop result in decreased nucleotide discrimination and fidelity and induce mutations [11], [12], [18], [19]. In addition, alterations in the amino acid sequence of the loop results in a reduced catalytic rate [12]. Pro242 is conserved between all members of the Pol X polymerase family [20], [21]. Prolines can cause rigidity in the protein structure and this characteristic may help to anchor this flexible loop. We previously proposed that the specific geometry of this loop, and particularly of residue 242, is important for maintaining the β-sheet structure of the Pol β active site. A change from Pro to Arg could disrupt the overall structure of the loop. Thus, it is not surprising to find that alteration of residue 242 from Pro to Arg results in an enzyme with low DNA polymerase activity.

### Accumulation of BER Intermediates Play a Role in Cellular Transformation Induced by P242R

Cancer is a disease of aging. We suggest that slow accumulation of genomic instability over 50–60 years may occur in people carrying the P242R germline variant and that genomic instability could lead to cancer. P242R catalyzes single nucleotide gap filling at a rate half that of WT Pol β, although the protein binds to DNA with similar affinity as WT Pol β (Figure 5). Because P242R is a slow polymerase, gaps and SSBs undergoing repair with P242R may accumulate at levels somewhat higher than those repaired by WT Pol β. Indeed, treatment of cells with MMS for 30 min leads to significantly increased SSB levels in cells expressing P242R versus WT (Figure 2A), even after the cells have had a chance to recover. At pH>12.6, the alkaline comet assay detects SSBs, BER intermediates from incomplete repair, and alkali-labile sites [20], [21], but others report that it also detect DSBs [22]. Monitoring of γH2AX under our conditions suggests the presence of few DSBs (Figure 2B). Our results suggest that SSBs and single nucleotide gaps accumulate in P242R-expressing cells, likely as a result of the slow gap filling activity of P242R.

Treatment of cells for 2 hours with MMS results in the appearance of greater levels of DSBs in cells expressing P242R versus WT Pol β, and we show that they form predominantly during S-phase. These results suggest after two hours of MMS treatment, the BER system becomes overwhelmed with DNA damage, resulting in fewer gaps being filled by Pol β and leading to DSB formation upon encounter of the replication fork by a gap. However, cells expressing WT Pol β recover from this treatment more quickly than cells expressing P242R. This is likely due to deficient gap filling by P242R. Our results are also consistent with the possibility that treatment with MMS for 2 hours induces frank DSBs that are more rapidly repaired by WT versus P242R Pol β. We do not favor this explanation because to date there is no evidence for Pol β having a direct role in the repair of DSBs. In addition, our cell cycle data suggests that the DSBs accumulate during S-phase, which is consistent with the idea that they originate as a result of replication of a break or gap in the DNA. This mechanism has been suggested to explain similar findings that have been reported in Pol β^−/−^ cells, where MMS treatment in G1 leads to unrepaired gaps that can form DSBs when cells enter G2/M [23]. Additionally, treatment of cells with a low dose of MMS and a PARP inhibitor, which effectively inhibits BER, resulted in DSB formation primarily during S-phase [24].

We have also shown that expression of P242R induces an elevated frequency of chromosomal aberrations compared to cells expressing WT Pol β (Figure 1). We suggest that these aberrations arise as the result of the persistent accumulation of BER intermediates in cultured cells (Figure 2), and that the presence of this type of genomic instability leads to cellular transformation (Figure 3). We show that cells expressing P242R undergo cellular transformation as demonstrated by formation of foci, anchorage-independent growth, and an increased rate of proliferation. The increase in proliferation suggests that critical cell cycle check points may be disrupted and cells are growing without effectively repairing the DNA. This uncontrolled cellular growth and replication of damaged DNA can induce the genomic instability we observe in these cells.

### Cells Expressing P242R Accumulate Endogenous DSBs

For many of the experiments we performed we increased the levels of DNA damage over endogenous levels by treating with MMS, which would be expected to increase the amounts of Pol β substrates, namely, single nucleotide gaps in cells, in order to be able to detect breaks and aberrations. However, we find that the levels of endogenous DSBs are increased in cells expressing P242R compared to cells expressing only WT Pol β (Figure 2B). Since Pol β is responsible for repairing at least 20,000 lesions/cell/day of endogenous damage, it is probable that humans expressing the P242R SNP may have more unresolved lesions compared to those with two WT alleles. Our finding of endogenous DSBs in cells expressing P242R suggests that over time, cells harboring this variant would incur more DSBs than cells without P242R even in the absence of exogenous DNA damage. We envision that the majority of DSBs are repaired accurately but that some of them are not, leading to deletions and insertions if repaired by non-homologous end joining, gene fusions, or other types of genomic instability, which could lead to cancer. Environmental exposures and/or diagnostic procedures over a P242R-carrying individual's lifetime could serve to enhance the rate or levels of genomic instability and perhaps decrease the latency of cancer.

### P242R and Cancer Therapy

Given that the P242R variant is rare (maximum allele frequency 2.4%) [6], epidemiologic studies have had limited success determining the role of this variant in human health [25]. It has been suggested that the 242Arg allele is associated with poor prognosis in lung cancer and lymphoma patients [9], [10], but the mechanism is unknown. One possibility is that the slow rate of DNA synthesis of P242R might be expected to enhance cancer cell death in the presence of DNA damage induced by radio- and chemotherapies, because many DNA gaps would remain unfilled. Indeed, we have shown that expression of P242R in Pol β-deficient MEFs does not rescue cells as completely as WT Pol β. This suggests that in the presence of P242R alone, for example in a homozygote who carries two alleles of P242R, would lead to cell death as a result of treatment with alkylating agents. However, expression of P242R in the presence of WT Pol β, as would be the case with a heterozygotic individual, only slightly sensitizes cells to high concentrations of MMS (Figure 4). This suggests that the majority of cells expressing both P242R and WT survive treatment with alkylating agents. We suggest that these survivors could have increased levels of genomic instability, based upon our results showing that when treated with MMS, cells expressing both P242R and WT Pol β accumulate BER intermediates and have an increased frequency of chromosomal aberrations. Thus, treatment of cells expressing both of these proteins could lead to cellular transformation or more aggressive disease.

In conclusion, our results show that the presence of the P242R germline variant contributes to the increase in chromosomal aberrations and cellular transformation. Therefore, individuals carrying this germline variant may have increased cancer susceptibility, suggesting that aberrant BER at the level of the germline could be a driver of carcinogenesis.

## Materials and Methods

### Chemicals and Reagents

All ultrapure deoxynucleoside triphosphates (dNTPs) were purchased from New England Biolabs. [γ-^32^P] ATP (5 mCi) and ATP were purchased from Amersham Biosciences and Sigma-Aldrich, respectively. All oligonucleotides used for the *in vitro* biochemical assays were purchased from Keck Biotechnology Research Center at Yale University and purified as described [26]. All oligonucleotides used for cloning and PCR were purchased from Invitrogen and are shown in Table S2.

### Plasmids and Cloning

Human Pol β cDNA (Genbank accession NM_002690) was cloned into the pET28a expression plasmid (Novagen) for expression with an N-terminal 6×His tag. WT Pol β cDNA sequence was verified by sequencing at the Keck DNA Sequencing Facility at Yale University. For cell culture experiments, human Pol β cDNA with a C-terminal hemagluttinin (HA) tag was cloned into the pRVYTet-Sis retroviral vector as described [14] [15]. The 242Arg variant was introduced into the human WT (242Pro) Pol β cDNA sequence using site-directed mutagenesis (Stratagene) following the manufacturer's protocols.

### Bacterial Strains, Mammalian Cell Lines, and Cell Culture

For cloning of Pol β, *E. coli* DH5αMCR with the genotype *mcrA* (*mrr-hsdRMS-mcrBC*)φ80Δ*lacZ*(M15) (*lacZYA-argF*)U169 *deoR recA1 endA1 phoA supE44 thi-1 gyrA96 relA1* was used. Human Pol β was expressed in Rosetta(DE3) cells (Novagen). For the λ*cII* forward mutagenesis assay, lysogen strain NM759 [27] was used for the preparation of sonication extracts and BHB2688 [28], [29] was used for the freeze-thaw extracts. *E. coli* strain G1250 *hflA::Tn5 hflB29* was used for selection and harvesting of packaged phage harboring mutations in the *cII* gene.

Mouse embryonic fibroblast (MEF) cell lines 92TAg (Pol β^+/+^) and 88TAg (Pol β^−/−^) were gifts from Leona Samson (Massachusetts Institute of Technology) [30], [31]. These cells were maintained in high-glucose Dulbecco modified Eagle's medium (Invitrogen) supplemented with 10% fetal bovine serum (Invitrogen), 1% penicillin-streptomycin (Invitrogen) and 1% L-glutamine and grown at 37°C in a 5% CO_2_ humidified incubator.

MCF10A cells are immortalized, non-transformed epithelial cells derived from human mammary tissue (ATCC). These cells were maintained in DMEM/F12 medium (Invitrogen) supplemented with 5% horse serum (Invitrogen), 1% penicillin-streptomycin, epidermal growth factor (20 ng/ml), hydrocortisone (0.5 µg/ml), cholera toxin (100 ng/ml), insulin (10 µg/ml) (Sigma-Aldrich) and grown at 37°C in a 5% CO_2_ humidified incubator.

C127 cells have been described [32]. The C127λ cells were made by a procedure similar to the one described in [28] with the following exceptions. The λsup-Fneo vector (kind gift from Dr. Peter Glazer, Yale University School of Medicine) was transfected into C127 cells using FuGene 6 (Roche). Single clones were selected in 900 mg/mL of G418 and expanded. A dot blot was used as described [28] to identify clones carrying the λsup-Fneo DNA. The C127λb clone was used in the experiments described here. C127λb cells were grown in Dulbecco modified Eagle's medium (Invitrogen) supplemented with 10% fetal bovine serum (Invitrogen), 1% penicillin-streptomycin (Invitrogen), and 600 µg/ml G418 at 37°C in a 5% CO_2_ humidified incubator.

The GP2-293 virus packaging cell line (Clontech) was used for retrovirus preparation. These cells were maintained in Dulbecco modified Eagle's medium (Invitrogen) supplemented with 10% fetal bovine serum (Invitrogen), 1% L-glutamine (Invitrogen), 1% penicillin-streptomycin (Invitrogen) and 1 mM HEPES (Invitrogen).

### Transfection, Infection, and Expression Analysis

Human Pol β WT and P242R constructs were packaged into retrovirus using the GP2-293 packaging line. pRVYTet and pVSV-G plasmids were co-transfected into GP2-293 cells using standard calcium phosphate transfection, cells were grown for 72 hours, and retrovirus was harvested.

To infect C127λb, 92TAg (Pol β^+/+^), 88TAg cells (Pol β^−/−^), and MCF10A, cells were grown to approximately 30% confluence and infected with retrovirus in the presence of 4 µg/ml polybrene. Cells were incubated overnight in fresh media with 4 µg/ml polybrene. For selection of pools, cells were split 1∶3 the day after infection and cells with the integrated construct were selected with 220 µg/ml hygromycin B for the C127λb and MEFs and 15 µg/ml hygromycin B for the MCF10A cells. For generation of stable clones, C127λb cells were split at several dilutions following infection and selected with 250 µg/ml hygromycin B. Single cell clones were grown and selected using cloning rings. Clonal cell lines were propagated in the presence of 160 µg/ml hygromycin B.

Expression of exogenous HA-tagged Pol β was verified by Western blot. Cells were passed in parallel in the presence or absence of tetracycline. Approximately 80–90% confluent cells were harvested by scraping with hot SDS Loading Buffer (50 mM Tris pH 6.8, 100 mM DTT, 2% SDS 10% glycerol). Lysates were boiled for 10 minutes and run on a 10% acrylamide SDS-PAGE gel. Proteins were transferred to nitrocellulose membrane using a semi-dry transfer apparatus and probed using monoclonal mouse anti-Pol β antibody (Abcam #1831).

### Genomic Instability Analysis

Four 10 cm dishes were seeded with 10^6^ cells per dish each per cell line and grown at 37°C 5% CO_2_ overnight. The cells were fed fresh medium and colcemid (Invitrogen) was added to a final concentration of 100 ng/ml. MCF10A pools were incubated in colcemid for three hours before harvesting by mitotic shakeoff. Cells were harvested via centrifugation, washed twice with 1× PBS, and resuspended dropwise in 0.8% sodium citrate. Following lysis, cells were incubated at 37°C for 30 minutes before fixing in Carnoy's Fixative (75% methanol, 25% acetic acid). Finally, cells were dropped onto microscope slides, dried and stained with 5% KaryoMax Giemsa stain (Invitrogen). Well-spread metaphases were identified under 100× objective (Zeiss). Images were taken using Spot Camera software (Diagnostic Instruments). Metaphase spreads were de-identified and scored by eye for chromosomal fusions, breaks, acentromeric chromosomes and fragments.

### Single-Cell Gel Electrophoresis Assay (Comet Assay)

Equal numbers of cells (4×10^5^) were plated in 60 mm dishes. The following day, the cells were treated with 2 mM MMS for 30 min. After treatment, the cells were prepared and analyzed immediately according to published procedures [33] using Cometslides (Trevigen Cat # 4250-200-03). Image analysis of 100–125 cells was performed using CometScore software (TriTek, Sumerduck, VA). Data are represented as mean ± SEM (n = ).

### Flow Cytometry

MCF10A cells expressing WT or P242R Pol β were untreated or treated with 2 mM MMS for 2 h. Cells were rinsed then with PBS and replaced with fresh media. Cells were allowed to recover for 0 h and 2 h post treatment. Cells were harvested by trypsinization, washed once with PBS, and pelleted. The pellet was resuspended by adding 70% ice cold ethanol dropwise while vortexing. Cells were fixed overnight at −20°C. The cells were incubated with primary phospho-γH2AX antibody (Millipore 05-636) 1∶500 overnight at 4°C. Following the incubation, cells were washed twice with PBS and incubated with anti-mouse secondary antibody conjugated to FITC 1∶500 for 1 h at room temperature. Cells were washed twiced with PBS and resuspended in 500 µl PI/RNase staining buffer (BD Pharmingen). Fluorescence was analyzed by flow cytometry using the BD FACSCalibur and analyzed using FlowJo 8.8.6 software.

### Cellular Transformation

The focus formation assay was conducted as in [15]. Briefly, cells were passaged every 3–4 days in the presence of 160 µg/ml hygromycin B and the presence (non-induced) or absence (induced) of 3 µg/ml tetracycline. Every four passages, 1×10 ^5^ cells were seeded into each of four T25 flasks. These cells were fed every 3–4 days with media containing or lacking 3 µg/ml tetracycline. After 21 days, the cells were stained with 0.25% crystal violet. The presence of foci was also monitored by microscopic examination as described previously [14], [15]. Anchorage independent growth was assessed as previously described [14]. Approximately 1×10^4^ MCF10A cells were mixed with media containing 0.7% noble agar (USB). This mixture was poured onto a layer of media containing 1.0% noble agar in a well of a 6-well dish. Cells were fed twice weekly for 4 weeks. The number of colonies present in each of five microscope fields per well from a total of 6 wells per experiment were counted after 4 weeks of growth.

### Clonogenic Survival

Cells (2.2×10^5^) from MEF pools were seeded into 60 mm dishes and incubated for 48 hours. Cells were treated with varying concentrations of MMS for two hours followed by trypsinization and plating at dilutions. Treated cells were grown for 9–11 days before staining with 0.25% crystal violet. Colonies were scored by eye at 4× magnification. Only colonies with more than 50 cells were counted and all experiments were repeated at least twice. For MCF10A cells, various concentrations of cells were plated in 6 well dishes and were allowed to attach overnight. Cells were treated with varying concentrations of MMS for two hours. Following treatment, cells were rinsed with PBS and replaced with fresh media. Cells were allowed to grow for 12–14 days before staining with crystal violet as detailed above.

### Cellular Proliferation

MCF10A pools were plated at a density of 25,000 or 50,000 cells per 10 cm dish. Cells were counted every day for 5 consecutive days. Data were plotted as change in cell number per day.

### Protein Expression and Purification

pET28a plasmids with human Pol β with 242Pro (WT) and 242Arg (242Arg) cDNA were transformed into Rosetta(DE3) cells. Protein expression was induced by addition of 1.0 mM isopropyl β-_D_-thiogalactopyranoside (IPTG) and grown at 37°C for 2 hours before harvesting via centrifugation. Cell pellets were dried and stored at −80°C. Protein induction was verified by using 10% SDS-PAGE stained with Coommassie Blue.

Protein was purified using fast protein liquid chromatography. Crude proteins were run through a HiTrap Chelating HP column (GE Healthcare) charged with Ni^2+^ using a linear imidazole gradient (from 5 to 500 mM) in 40 mM Tris-HCl pH 8.0, 500 mM NaCl. The His-fusion proteins eluted at approximately 277 mM imidazole over five or six fractions of two ml each. The fractions were combined, concentrated to less than one milliliter and diluted into nine milliliters low salt buffer (50 mM Tris-HCl pH 8 .0, 1 mM EDTA, 10% glycerol, 0.1 M NaCl). The diluted protein was then run on a SP HP column (GE Healthcare) using a linear NaCl gradient from 100 mM to 2000 mM. Purified protein fractions eluted at approximately 1000–1200 mM NaCl in two fractions, which were combined and concentrated to less than one milliliter. Glycerol was added to a final concentration of 10–15% and aliquots were flash frozen in liquid N_2_ and stored at −80°C. All proteins were purified to >90% homogeneity based on Coomassie Blue staining of 10% SDS-PAGE gels.

### Preparation of DNA Substrates

Oligonucleotides were synthesized by the W.M. Keck facility and purified by polyacrylamide gel electrophoresis as described previously [34]. Briefly, primer oligos were radiolabelled with γ-^32^P ATP using T4 polynucleotide kinase (New England Biolabs) and downstream oligos were kinased using non-radioactive ATP. Kinased oligonucleotides were purified using Microspin columns (Biorad). Primer, template, and downstream oligos were annealed by denaturing at 95°C for 5 minutes, slow cooling to 50°C for 30 minutes, holding at 50°C for 20 minutes, and then resting on ice. Complete substrate annealing was confirmed using 12% native polyacrylamide gel electrophoresis and visualized using autoradiography.

### Pre-Steady State Burst

Radiolabeled 1 bp gapped DNA (300 nM 45AG [35]) and Pol β (100 nM) were combined with the correct dNTP and 10 mM MgCl_2_ in a KinTek Chemical Quench-Flow apparatus at 37°C. The reactions were quenched by the addition of 0.5 M EDTA. The reaction products were separated on a 20% denaturing polyacrylamide gels, visualized, and quantified using a Storm 860 Phosphorimager with ImageQuant software. Data were fitted to the burst equation:
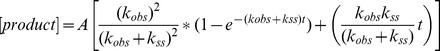
where A is the amplitude, *k*_obs_ is the observed rate constant of the exponential phase, and *k*_ss_ is the rate constant of the linear phase [36].

### Gel Electrophoretic Mobility Shift Assay

The DNA binding constant was determined by gel electrophoretic mobility shift assay as described previously [34]. The dissociation constant for DNA (K_D_) was determined by fitting the fraction bound protein (Y) versus protein concentration with the equation:
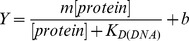
wherer Y is the amount of bound protein, m is a scaling factor, and b is the apparent minimum Y value [37].

### λcII Forward Mutagenesis Assay

High molecular weight DNA was harvested from C127λb cells using standard phenol chloroform extraction follwed by dialysis against TE buffer as previously described [16]. Sonication and freeze-thaw packaging extracts were prepared as in [28], [29] using NM759 and BHB2688 *E. coli* strains. To rescue the λ vector from the DNA, approximately 5 µg of high molecular weight genomic DNA was added to 60 µl of sonication extracts mixed with 40 µl of freeze-thaw extracts. Reactions were incubated at 32°C for 90 minutes. An additional 100 µl of packaging extracts was added and the reactions were incubated for an additional 90 minutes at 32°C. For cII gene mutation detection, the *in vitro* packaged phage were diluted, adsorbed in G1250 bacteria, and plated in 0.4% top agar on TB plates. Plates were incubated at 37°C overnight to determine the infection titre, because due to a termperature-sensitive mutation in the cII gene in the phage vector, all phage are lytic and form plaques at this termperature. Plates were also incubated at 24°C. The WT phage do not form plaques at this temperature whereas phage with mutations in the cII gene will. cII mutants were obtained from at least three independent packages. Mutant plaques were replated in fresh cultures. Phage DNA was harvested and used as a template for PCR. The cII genes were amplified by PCR and sequenced at the W.M. Keck Sequencing facility.

### Statistics

Two-tailed t-tests and two-way analysis of variance (ANOVA) were used as appropriate to determine whether the mean of each cell line was different from the empty vector cells. Bonferroni's post hoc test was used to determine significant differences between the means of each group. All statistics were performed using GraphPad Prism version 5 (GraphPad Software, San Diego, CA). Data are represented as mean ± SEM.

## Supporting Information

Figure S1 Exogenous Pol β expression in cell lines. A. Representative western blot for Pol β expression in human MCF10A cells. Lane 1 is from the WT line and lane 2 is from the P242R line. The ratio of exogenous to endogenous is shown below the blot. B. Representative western blots for Pol β expression in C127λb clonal cell lines. Arrow points to band for HA-tagged exogenous Pol β. Lysates from cells grown in the presence of tetracycline, meaning that exogenous Pol β is not expressed, were run in odd numbered lanes. Lysates from cells grown in the absence of tetracycline were run in even numbered lanes. Lanes 1 and 2 are from WT8 cell line, lanes 3 and 4 from WT20, lanes 5 and 6 from P242R5, and lanes 7 and 8 from P242R15. Cell lines are the same as those used in the cellular transformation assays. The WT8 and 20 clones express WT Pol β exogenously and the P242R5 and 15 express the P242R protein exogenously.
(TIFF)

Figure S2 λ*cII* mutation spectrum. The wild type sequence of the *cII* gene is shown in black with the corresponding base numbers on the right. Mutations identified in cells not expressing the P242R variant of Pol β are shown above the wild type sequence in red. Mutations identified in cells expressing the P242R variant are shown below the wild type sequence in blue. Single letters represent base substitutions, open triangles represent single nucleotide deletions, filled triangles represent single nucleotide insertions, and open diamonds represent deletions in repeated sequence.
(TIFF)

Figure S3 γH2AX immunofluorescence in MEFs following MMS treatment. A. Representative cell expressing WT Pol β before MMS treatment. B. Representative cell expressing P242R Pol β before MMS treatment. C. Representative cell expressing WT Pol β immediately following 2 hour exposure to 0.25 mM MMS. D. Representative cell expressing P242R Pol β immediately following 2 hour exposure to MMS. E. Representative cell expressing WT Pol β 4 hours following MMS exposure. F. Representative cell expressing P242R Pol β 4 hours following MMS exposure. G. Percentage of cells with high γH2AX staining before (No MMS), immediately following (0 min), and at indicated timepoints following exposure to 0.25 mM MMS for 2 hours. At least 50 cells were analyzed per treatment.
(TIFF)

Table S1 Summary of λcII forward mutation assay. Raw numbers (# mut.) and mutation frequency (M.F.) for each type of base mutation are shown for cells not expressing (not induced) or expressing (induced) exogenous P242R Pol β.
(TIFF)

Table S2 Details of oligonucleotides used for cloning and PCR.
(DOC)
