# Supplementary figures and images for: A Germline Polymorphism of DNA Polymerase Beta Induces Genomic Instability and Cellular Transformation
Source: PLoS Genet. 2012 Nov 8;8(11):e1003052. doi: 10.1371/journal.pgen.1003052 (PMC3493456; doi:10.1371/journal.pgen.1003052)

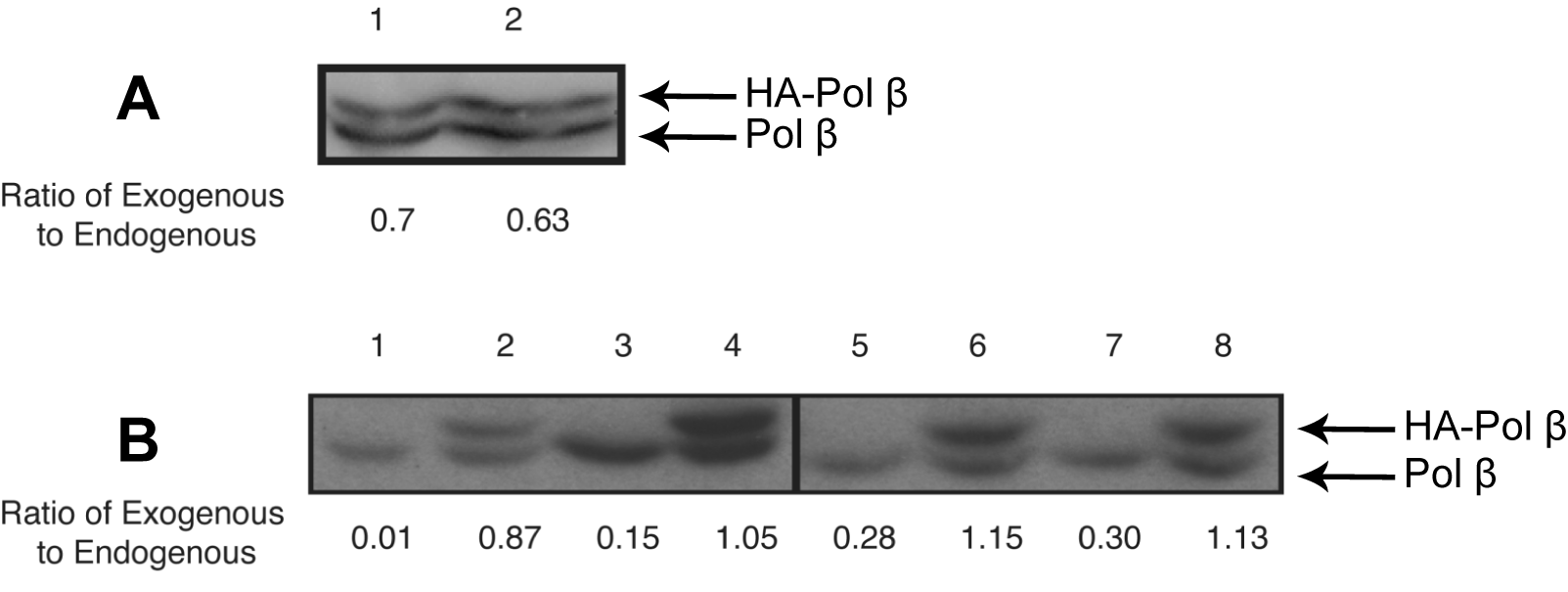

Supplement: Figure S1 — Exogenous Pol β expression in cell lines. A. Representative western blot for Pol β expression in human MCF10A cells. Lane 1 is from the WT line and lane 2 is from the P242R line. The ratio of exogenous to endogenous is shown below the blot. B. Representative western blots for Pol β expression in C127λb clonal cell lines. Arrow points to band for HA-tagged exogenous Pol β. Lysates from cells grown in the presence of tetracycline, meaning that exogenous Pol β is not expressed, were run in odd numbered lanes. Lysates from cells grown in the absence of tetracycline were run in even numbered lanes. Lanes 1 and 2 are from WT8 cell line, lanes 3 and 4 from WT20, lanes 5 and 6 from P242R5, and lanes 7 and 8 from P242R15. Cell lines are the same as those used in the cellular transformation assays. The WT8 and 20 clones express WT Pol β exogenously and the P242R5 and 15 express the P242R protein exogenously. (TIFF) [file pgen.1003052.s001.tif]

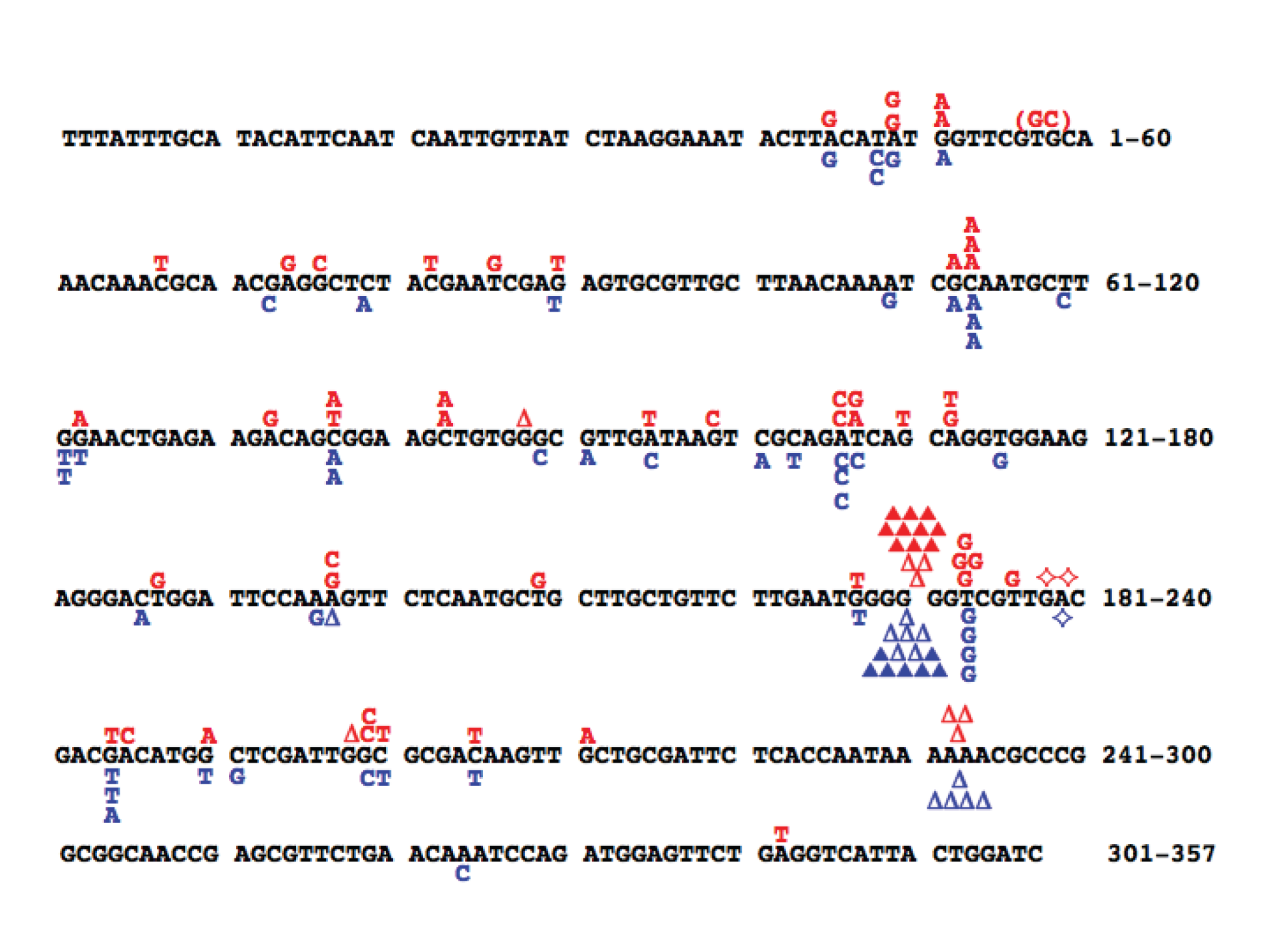

Supplement: Figure S2 — λcII mutation spectrum. The wild type sequence of the cII gene is shown in black with the corresponding base numbers on the right. Mutations identified in cells not expressing the P242R variant of Pol β are shown above the wild type sequence in red. Mutations identified in cells expressing the P242R variant are shown below the wild type sequence in blue. Single letters represent base substitutions, open triangles represent single nucleotide deletions, filled triangles represent single nucleotide insertions, and open diamonds represent deletions in repeated sequence. (TIFF) [file pgen.1003052.s002.tif]

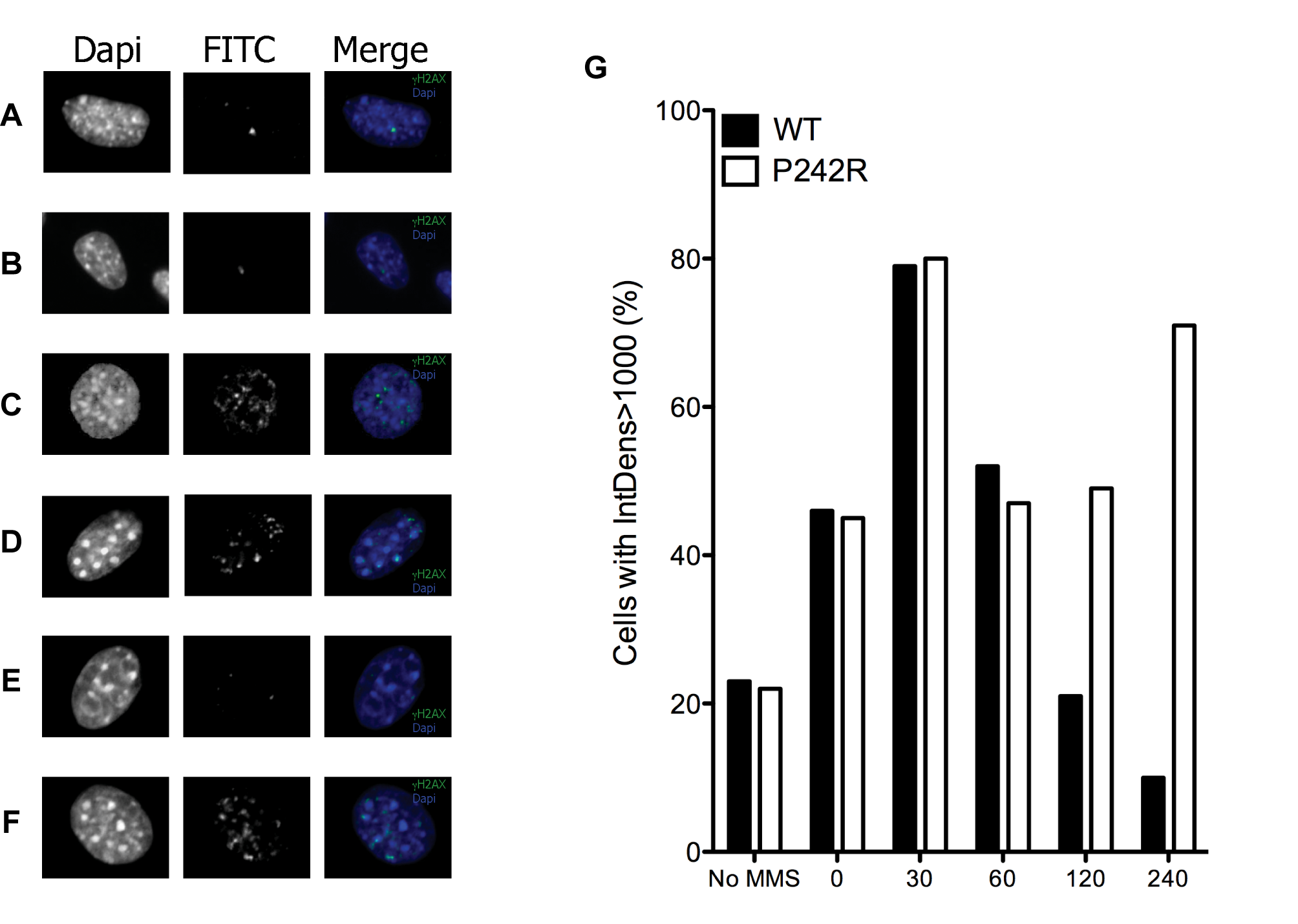

Supplement: Figure S3 — γH2AX immunofluorescence in MEFs following MMS treatment. A. Representative cell expressing WT Pol β before MMS treatment. B. Representative cell expressing P242R Pol β before MMS treatment. C. Representative cell expressing WT Pol β immediately following 2 hour exposure to 0.25 mM MMS. D. Representative cell expressing P242R Pol β immediately following 2 hour exposure to MMS. E. Representative cell expressing WT Pol β 4 hours following MMS exposure. F. Representative cell expressing P242R Pol β 4 hours following MMS exposure. G. Percentage of cells with high γH2AX staining before (No MMS), immediately following (0 min), and at indicated timepoints following exposure to 0.25 mM MMS for 2 hours. At least 50 cells were analyzed per treatment. (TIFF) [file pgen.1003052.s003.tif]

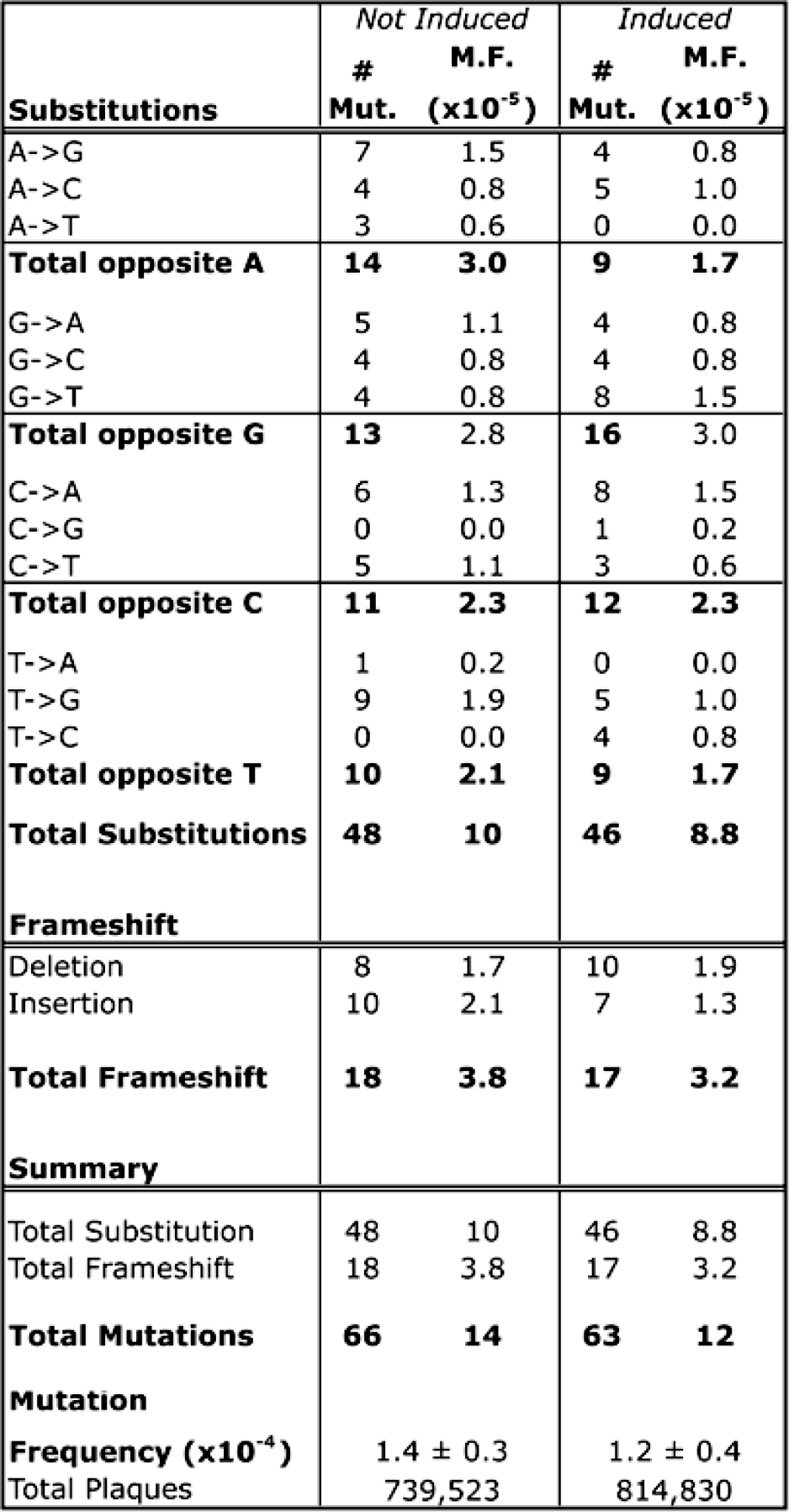

Supplement: Table S1 — Summary of λcII forward mutation assay. Raw numbers (# mut.) and mutation frequency (M.F.) for each type of base mutation are shown for cells not expressing (not induced) or expressing (induced) exogenous P242R Pol β. (TIFF) [file pgen.1003052.s004.tif]
