# Supplementary material for: A Germline Polymorphism of DNA Polymerase Beta Induces Genomic Instability and Cellular Transformation
Source: PLoS Genet. 2012 Nov 8;8(11):e1003052. doi: 10.1371/journal.pgen.1003052 (PMC3493456; doi:10.1371/journal.pgen.1003052)
Supplement: Table S2 — Details of oligonucleotides used for cloning and PCR. (DOC) [file pgen.1003052.s005.doc]

| **Sequence (5’-3’)** | **Experimental Use** |
| --- | --- |
| CAG CAT ATG AGC GAA ATG AGC AAA CGG AAG | pET28a cloning forward |
| CAG GCG GCC GCT CAT TCG CTC CGG TCC TTG GGT TC | pET28a cloning reverse |
| TTA GCG GCC GCAT GAG CAA ACG GAA GGC | pRVY cloning forward |
| TAA GGA TCC TCA TTC GCT CCG GTC C | pRVY cloning reverse |
| GTT CAT GGG TGT TTG CCA GCT TC**G** CAG TAA AAA TGA TGA AAA AG | P242R site-directed mutatgenesis forward |
| CTT TTT CAT CAT TTT TAC TG**C** GAA GCT GGC AAA CAC TGA AC | P242R site-directed mutatgenesis reverse |
| 5’-GCCTCGCAGCCGTCCAACCAAC CAACCTCGATCCAATGCCGTCC- 3’  3’-CGGAGCGTCGGCAGGTTGGTTG**A**GTTGGAGCTAGGTTACGGCAGG- 5’ | Pre-steady state kinetics, gel mobility shift assay |
